# Electrochemical Liquid Phase TEM in Aqueous Electrolytes for Energy Applications: the Role of Liquid Flow Configuration

**DOI:** 10.1002/smtd.202401718

**Published:** 2024-11-27

**Authors:** Katarzyna Bejtka, Marco Fontana, Cecilia Irene Gho, Stefan Merkens, Andrey Chuvilin, Candido Fabrizio Pirri, Angelica Chiodoni

**Affiliations:** ^1^ Department of Applied Science and Technology Politecnico di Torino Corso Duca degli Abruzzi 24 Torino 10129 Italy; ^2^ Center for Sustainable Future Technologies @Polito Istituto Italiano di Tecnologia Via Livorno 60 Torino 10144 Italy; ^3^ Electron Microscopy Laboratory CIC nanoGUNE BRTA Tolosa Hiribidea 76 Donostia‐San Sebastian 20018 Spain; ^4^ Ikerbasque Basque Foundation for Science Bilbao 48013 Spain

**Keywords:** catalysis, CO2RR, electrochemistry, energy transition, in situ liquid phase TEM

## Abstract

Electrochemical liquid phase transmission electron microscopy (EC‐LPTEM) is an invaluable tool for investigating the structural and morphological properties of functional materials in electrochemical systems for energy transition. Despite its potential, standardized experimental protocols and a consensus on data interpretation are lacking, due to a variety of commercial and customized electrical and microfluidic configurations. Given the small size of a typical electrochemical cell used in these experiments, frequent electrolyte renewal is crucial to minimize local chemical alterations from reactions and radiolysis. This study explores the effects of modifying the flow configuration within the liquid cell under experimental conditions relevant for energy applications in aqueous‐based electrolytes, revealing how changes in mass transport dynamics drastically influence the electrochemical response of the cell. Two different cell designs are compared: convection‐ and diffusion–governed. Ex situ and in situ comparative flow experiments show that the *diffusion cell* mitigates gas bubbles formation *and* improves removal of gaseous products. The electrodeposition of Zn nanostructures and the characterization of a Cu‐based catalyst are presented as proof‐of‐concept experiments for energy storage and CO_2_ reduction reaction (CO2RR) applications, respectively. The reported findings demonstrate that controlling mass transport in the liquid cell setup is crucial to obtain reliable operando experimental electrochemical conditions.

## Graphical abstract

The implications of the *diffusion cell* design for electrochemical liquid phase TEM are explored in relevant conditions for energy applications in aqueous electrolyte. The crucial role of the liquid flow configuration on the electrochemical response of the cell is shown through specifically designed tests and two proof‐of‐concept experiments: electrodeposition of Zn nanostructures and electrochemical reduction of CO_2_.

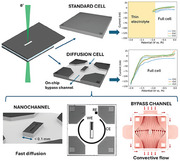

## 1 Introduction

In recent years, transmission electron microscopy (TEM) in liquid environment with controlled liquid flow has become possible through the development of specific TEM holders based on a miniaturized liquid cell, directly exposed to the electron beam.^[^ ^1^ ^]^ The dramatic improvement in micro and nano fabrication techniques has led to liquid phase TEM (LPTEM) holders with multiple functionalities, such as mixing different solvents, temperature control, electrochemical stimulation.^[^ ^2^, ^3^ ^]^ In fact, these features open, in principle, a broad range of exciting investigation possibilities for fundamental as well as applied research, allowing direct materials imaging and correlated characterization of chemical and physical properties at the nanoscale in liquid environment. However, LPTEM poses many experimental challenges, since the liquid environment is responsible for resolution decrease and loss of contrast in TEM/STEM imaging and electron diffraction^[^ ^4^, ^5^ ^]^ compared to the vacuum environment. Moreover, the interaction between the electron beam and the liquid in the cell gives rise to unwanted side chemical reactions,^[^ ^6^ ^]^ local pH and pOH variations^[^ ^7^ ^]^ and bubbles formation,^[^ ^8^ ^]^ especially in aqueous solutions. Notwithstanding the afore‐mentioned limitations, liquid cell TEM can have a dramatic impact in studying electrochemically‐driven phenomena at the nanoscale, by making use of the electrochemical functionality developed in LPTEM holders. This technique is usually referred to as in situ electrochemical liquid phase TEM (EC‐LPTEM). With this particular experimental set‐up, it is possible to perform electrochemical experiments in a three‐electrode configuration (working electrode WE, counter electrode CE, reference electrode RE) with simultaneous TEM/STEM characterization over the electron‐transparent WE region with a flowing electrolyte.

Li‐based batteries have been benefiting a lot of this innovative characterization approach and the first commercial technological solutions have been optimized to match the experimental need in this scientific framework.^[^ ^9^, ^10^, ^11^, ^12^ ^]^ In more recent years, electrocatalysis has been raising a lot of interest and the experimental set‐up has been adapted to match the different experimental needs as well.^[^ ^13^ ^]^

Among the various possible electrochemical applications, catalytic reactions for energy transition (hydrogen evolution reaction (HER), carbon dioxide reduction reaction (CO2RR), oxygen reduction reaction (ORR), oxygen evolution reaction (OER)) have been extensively studied in recent years, due to the growing impact of the global energy issue. EC‐LPTEM studies could provide deeper understanding of the fundamental mechanisms involved in the mentioned energy‐related reactions, resulting in rationally designed catalysts with increased catalytic activity, selectivity and stability.^[^ ^14^ ^]^ In the context of catalysis, one of the main challenges is to understand the morphological, structural and chemical modifications involved in the activation of the as‐prepared catalyst, which can be in the form of inactive pre‐catalyst,^[^ ^15^ ^]^ or more in general variations of the surface structure, amorphization processes, formation or loss of defective sites, dissolution and/or precipitation phenomena. It is also important to recognize whether these modifications occur during the first stages of the catalytic activity or after an extended period of time, affecting the stability and durability of the catalyst.^[^ ^16^, ^17^ ^]^ Most of these reactions involve the use of water‐based electrolytes, due to high ionic conductivity and reduced costs coupled with their safety, low toxicity and environmental friendliness, according to green chemistry principles.^[^ ^18^ ^]^

As mentioned above, besides catalytic applications, in situ EC‐LPTEM has also been applied in the broad field of energy storage, particularly in the context of electrochemical batteries.^[^ ^19^ ^]^ Research studies are needed to clearly correlate the effect of crystal structure, particle aggregation and agglomerate size on the electrochemical properties. These properties are probed during the electrochemical stimulation in order to understand their correlation and dynamical evolution. The information obtained from these studies can be of high importance for designing energy storage systems with higher energy and power densities coupled with excellent stability.^[^ ^20^ ^]^ It is foreseen that the gold‐standard lithium‐ion battery will compete with aqueous batteries (with water‐based electrolytes) generally regarded as safe, reliable and affordable.^[^ ^21^ ^]^

It has been demonstrated till now how the mentioned applications for energy transition can benefit from this challenging characterization approach. For all of them, there are some specific key aspects that characterize EC‐LPTEM experiments: i) the evolution of chemical species (gas/liquid) at the WE (on the electron‐transparent viewing window), ii) the evolution of reaction products at the CE when it experiences polarization, iii) radiolysis products generated by the electron beam.^[^ ^22^ ^]^

All the afore‐mentioned effects dynamically alter the (electro‐)chemical framework inside the miniaturized liquid cell, diminishing the experimental control and limiting the interpretation of the experimental results. Mitigation of these effects thus is a crucial task to advance EC‐LPTEM into a quantitative and reliable technique.

Since radiolysis effects are driven by the electron beam, possible mitigation approaches rely on minimizing the electron dose.^[^ ^23^ ^]^ The evolution of chemical species at the WE and at the CE is primarily driven by the electrochemical conditions; consequently, mitigation strategies may rely on a reaction‐dependent fine tuning of the electrochemical testing conditions. Moreover, efficient solution renewal, based on optimized design of the miniaturized liquid cell is a comprehensive mitigation strategy for both radiolysis^[^ ^24^ ^]^ and electrochemical effects. The implementation of mitigation strategies is of paramount importance, especially in aqueous electrolytes, which usually involve the water splitting reaction (as main or side reaction) at the electrodes interface. For example, in CO2RR studies, this is a typical side reaction arising when the WE is kept at negative potentials versus RHE, which causes the formation of H_2_ and O_2_ at the WE and CE respectively from water.^[^ ^25^ ^]^ Once the concentration of these gaseous products exceeds the solubility limit in water, gas bubbles are formed. Due to the limited (nanoliters to microliters range) volume of the liquid cell, the formation of gas bubbles quickly causes dewetting of the WE and ultimately causes the cell filling with gas which, in most of the cases, leaves a very thin electrolyte layer (< 50 nm) on the catalyst surface.^[^ ^26^ ^]^ While this phenomenon can be used to momentarily enhance the TEM resolution in specific situations,^[^ ^27^ ^]^ it is under debate whether the electrochemical stimulation in the so‐called “thin electrolyte” condition is actually comparable to a “bulk” electrolyte condition. Severe bubble formation is observed when negative or positive potential, depending on the reaction of interest, are applied over certain limits. As an example, at potentials which are relevant for *operando* studies of CO2RR, impacting bubble formation is observed with potentials more negative than −0.2 V versus RHE, therefore limiting the capabilities of the technique for this particular application, to the “thin electrolyte” condition.^[^ ^28^ ^]^ Thus, efficient removal of gaseous and liquid species may be regarded as the crucial challenge to advance from in situ to *operando* ^[^ ^29^ ^]^ approach.

Solution renewal, and therefore gaseous / liquid species removal, and provision of the fresh electrolyte, is driven by a combination of diffusion and convection, with the dominant mass transport mechanism depending on the microfluidic geometry. Specific architectures of EC‐LPTEM holders may favor one mechanism over the other.

In the “direct flow” systems the liquid flow is directed across the imaging area, local flow velocities up to 5·10^−2^ ms^−1^ were demonstrated, thus maximizing convective solution renewal.^[^ ^30^ ^]^ Such high flow velocities are, however, not always desired as they might lead to: i) increased operating pressure gradients ii) detachment of the sample from the viewing window, iii) detrimental effects during either imaging or electron diffraction acquisitions.^[^ ^30^ ^]^

In the so‐called “bathtub” design, in which a MEMS‐based liquid flow cell is embedded into an oversized cavity leaving space for the fluid to bypass the nanochannel inside the cell, the dominant mechanism is diffusion.^[^ ^31^ ^]^ It was demonstrated that the solution renewal dynamics in the central part of the viewing area, which is where in the EC applications the WE is positioned, can be as slow as tens of minutes,^[^ ^31^ ^]^ as a consequence of the large diffusion length.

The importance of diffusion for solution exchange was underlined in the literature with focus on the necessity for short distance between reservoirs of fresh solution (decreasing the diffusion length) to the viewing area.^[^ ^32^ ^]^ Recently, the *diffusion cell* concept was introduced, demonstrating enhanced solution renewal in the viewing area in this novel design, while avoiding high local flow velocities across the WE/viewing window, which could lead to unwanted sample removal.^[^ ^33^ ^]^

**Figure 1:**
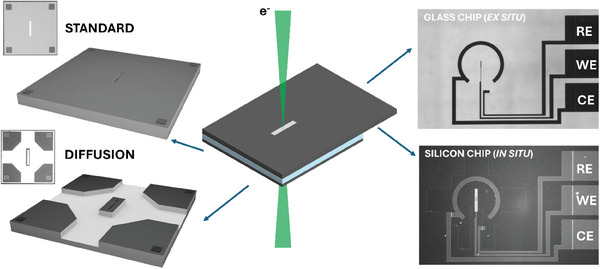
Exploded view of the electrochemical cell used to perform the EC‐LPTEM experiments. On the left side, the two small chip designs tested in the present work (*standard* and *diffusion*). On the right side, the glass and the silicon large top chips are shown, which were used in this work for ex situ and in situ experiments, respectively.

In the *diffusion cell* geometry, an on‐chip bypass channel is inserted in the small chip, while keeping the electron‐transparent viewing window on an elevated “island” in the center of the cell (see **Figure** **1**). In this way, the characteristic time‐scale of solution renewal is reduced in the viewing area, without the possible detrimental effects observed when applying high flow rates for the same purpose, since the contribution from convection is mainly confined outside the viewing area. This design influences mass transport conditions, enhancing diffusion in the viewing window and confining convective flow outside the viewing window.

In this work, we systematically investigate the implications of the *diffusion cell* concept for EC‐LPTEM experiments in aqueous electrolyte, in conditions which are relevant for applications related to the energy transition. The role of the liquid flow configuration is carefully examined, by means of specifically‐designed EC‐LPTEM experiments supported by predictions from fluid‐dynamics numerical models.

Two different microfluidic cell concepts are studied: i) the *standard* nano‐cell with planar geometry and ii) the *diffusion* cell with on‐chip by‐pass channel.

We particularly focus on the capability of reaction products removal from the electrochemical cell, taking into consideration the viewing area (WE region) and the CE region. The practical goal is to demonstrate an extended potential range for electrochemical experiments in aqueous electrolyte without significant accumulation of gas bubbles and indirectly efficient provision of fresh electrolyte. In the context of energy transition, we selected CO_2_ electroreduction and rechargeable Zn‐air batteries experiments to show the improved experimental control due to the optimized experimental set‐up.

## 2 Results and Discussion

The performance of the *diffusion* cell for electrochemical applications was evaluated and compared to the standard configuration through a series of comparative flow experiments. In order to validate potential benefits of the modified cell design for different in situ */ operando* EC‐LPTEM experiments, tests were conducted using both Pt and glassy carbon working electrodes. No additional catalytic material was involved in these experiments since the aim is to investigate the response of the liquid cell to electrochemical stimulation regardless of the specific material under study. This is particularly relevant since in most practical experiments the majority of the WE area is not totally covered by the catalyst, especially in those cases where the catalysts are drop‐casted on the WE prior to the experiment.^[^ ^34^, ^35^ ^]^

Regarding gas bubble formation, it is important to further stress on the fact that gaseous reaction products are not solely generated at the WE but also at the CE during electrochemical experiments. Therefore, it is important to monitor the generation of gaseous products over the whole cell. However, in situ TEM experiments provide direct information only from the WE that is placed in the electron‐transparent region. The CE is unfortunately out of view. To have a clear view of the whole cell during electrochemical stimulation, ex situ comparative flow experiments were conducted using customized optically‐transparent glass chips (Figure 1). The glass chips share the same size and electrode design as the standard silicon large chips, with a three platinum electrodes configuration. The choice of platinum as electrode material was particularly appropriate for these experiments, because it maximizes the production of gaseous H_2_ at the WE,^[^ ^36^ ^]^ allowing for easier comparison of the performance of the different flow configurations under the optical microscope. The full investigation of the cells with both the standard and the modified small chips was performed at three different flow rates (300, 1200, and 2500 µL h^−1^) of the 0.1 M KHCO_3_ aqueous electrolyte, which was previously saturated with CO_2_.

### 2.1 Ex Situ Comparative Flow Experiments

**Figure 2:**
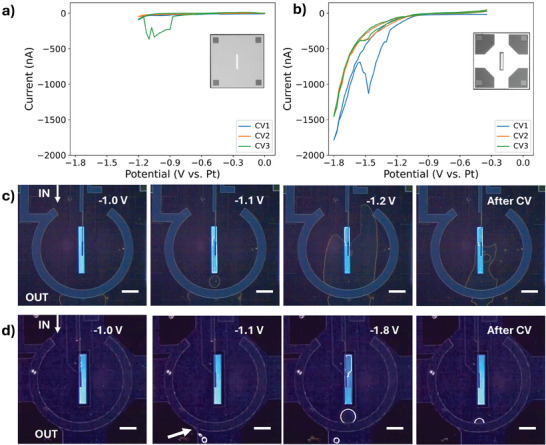
a) CVs in the range −1.2 V to 0 versus Pt (standard configuration) and b) −1.8 V to 0 V versus Pt (optimized configuration) with 1200 µL h^−1^ flow rate. Optical microscopy images are provided, extracted from the videos acquired during electrochemical stimulation for the standard c) and optimized d) cell. The scale bar is 200 µm.

**Figure** **2** provides a direct visualization of the liquid cell response to the electrochemical stimulation at potentials which are relevant for energy applications. Thanks to the optically‐transparent glass large chips, images and videos of the WE, RE and CE were obtained by optical microscopy. Based on the optical images, the main difference between the two cell geometries is immediately clear. In the standard configuration, the three electrodes all lie in the nanochannel, whose thickness is defined by the 500 nm spacer on the large chip and by the 150 nm spacer of the small chip (total thickness 650 nm). In the *diffusion* cell configuration, the WE is placed on the electron‐transparent “island” in the nanochannel, while the RE and CE are in the by‐pass channel (which is 10 µm thick). Since the electrolyte volume in the direct proximity of the CE is more than one order of magnitude larger in the modified configuration than in the standard one, it is expected that the cell would be able to sustain a stronger production of gaseous reaction products at the CE before the solubility limit is reached and bubbles start forming. This may also partially be true for the WE: although it lies in the nanochannel, the “island” is only 50 µm wide, while the region nearby (part of the by‐pass channel) provides a higher volume of electrolyte, possibly facilitating bubble dissolution. Finally, based on previously published numeric convection diffusion simulations, the optimized configuration enhances convective mass transport in the on‐chip bypass channel, which results in a more efficient removal of gas and liquid products promoting renewal of solution (also in the central nanochannel).^[^ ^33^ ^]^

The behavior of the standard flow configuration with 1200 µL h^−1^ flow rate during cyclic voltammetry at potentials between 0 V and −1.2 V versus Pt is shown in the Movie S1 (Supporting Information), while significant frames are reported in Figure 2c. As can be observed, the bubble formation phenomenon begins at the counter electrode interface at ≈ ‐1 V versus Pt, where gaseous O_2_ is produced. Since the CE is immersed in a sub‐micrometer‐thick layer of electrolyte, the solubility limit is easily reached and bubbles are formed. It is observed that the bubbles are not efficiently removed, instead they coalesce and continue to expand. At more negative potentials, they also merge with the bubbles formed by the H_2_ produced at the WE, forming a gas chamber at −1.2 V versus Pt which does not disappear immediately when the potential is set back to Open Circuit Potential (OCP, “after CV” in Figure 2). This behavior is reflected also by the CV curves (Figure 2a), showing unpredictable measured values of the current. After the last voltammetry cycle, the cell requires almost 10 min to completely recover the liquid electrolyte. It must be stressed that a similar behavior is observed at 1200 µL h^−1^ and 2500 µL h^−1^ flow rates, in the same range and also when the tests are performed in a wider potential range (enlarging the potential window to more negative potentials). At the flow rate of 300 µL h^−1^, the bubbles are not efficiently brought away, and they keep expanding already during the test in the range between 0 V and −1.0 V versus Pt.

Differently from the above discussed results, with the *diffusion* cell configuration it is possible to explore more negative potential values (down to −1.8 V vs. Pt, see Movie S2, Supporting Information) due to the larger available electrolyte volume and to the optimized liquid flow around the viewing window. The improved geometry and increased mass transport (through convection) in the by‐pass channel from one hand minimize the coalescence of gaseous reaction products, and from the other hand efficiently remove gas bubbles when they are formed.

The dynamics of gas bubbles inside this cell during a CV acquisition between 0 V and −1.8 V versus Pt at 1200 µL h^−1^ flow rate are presented in Figure 2d. Also in this case, the bubble formation process starts at the counter electrode, but its position inside the 10 µm bypass channel prevents the gas saturation. This assumption is confirmed by the observation of the gas bubbles formed at the position where the CE overlaps with the pentagonal plateau on the bottom left (second frame, Figure 2d): here, the thickness is locally reduced to 650 nm as in the standard cell condition. It is interesting to notice that the gas bubbles are readily brought away by the liquid flow. Concerning the WE region, it must be noted that at −1.1 V versus Pt the improved liquid flow configuration inhibits the formation of gas bubbles in the viewing window. However, when this process is boosted by more negative potentials, gaseous products form and coalesce in the WE region.

Since the WE is placed in front of the “island” of the small chip (650 nm channel thickness), it is expected to experience saturation of the gaseous products at milder conditions compared to the CE. Despite this, it still benefits from the improved diffusion transport given by the presence of the bypass channel around the “island”, effectively reducing the diffusion length. The contribution of diffusion to the overall enhanced removal of the products of reactions in this type of flow reactor is confirmed by convection diffusion modeling of solute removal, as shown in Note S4 (Supporting Information). The presence of bubbles on the WE does not cause complete dewetting of the WE, as they are easily moved to the bypass channel toward the outlet port (at the bottom of the optical images in Figure 2d) thanks to the favorable configuration of convective flow in the *diffusion* cell. In this regard, the first voltammogram (CV1) in Figure 2b is exemplary: while the formation of gas bubbles causes a sudden decrease of the current intensity at ≈ −1.45 V versus Pt, the contact between the electrolyte solution and the WE is not completely lost. Consequently, it is possible to acquire consecutive cyclic voltammograms with comparable current response (see CV2, CV3).

Due to the improved management of bubbles inside the cell, with this optimized configuration only 1 min (ten times faster than the standard configuration) is needed to completely recover the electrolyte in cases where the WE region suffers partial dewetting.

The modified liquid flow configuration results in improved efficiency for the removal of gaseous products at all tested flow rates when the cyclic voltammetry is run in the potential range up to −1.4 V versus Pt. For the flow rate of 300 µL h^−1^, at more negative potentials the gas products formed accumulate into the bubbles, which coalesce before being efficiently removed. Complete dewetting of the WE does not occur, and the bubbles are removed partially when the cyclic voltammetry returns to less negative potentials. The behavior for the flow rate of 2500 µL h^−1^ is in good agreement with the one at 1200 µL h^−1^ flow rate.

The ex situ comparative flow experiments allowed to visualize the dynamics of bubbles in the whole cell and, in particular, they were used to analyze the ability of the by‐pass channel to remove gaseous products.

As a final remark on the ex situ experiments, the pivotal role of the custom‐made optically transparent large glass chips must be highlighted. The possibility of visualizing through optical microscopy the entire liquid cell is extremely important to understand the cell behavior under electrochemical stimulation. This kind of investigation might be envisioned as a preliminary step in the optimization of the experimental conditions for carefully planned EC‐LPTEM experiments. The influence of the experimental conditions (flow channel cross‐section, flow rate, potential window) on the electrochemical response of the whole liquid cell might thus be studied before the in situ experiments in the TEM column.

### 2.2 In Situ Comparative Flow Experiments

As previously discussed, ex situ comparative flow experiments provide direct visualization of the effects of the optimized flow configuration for electrochemical experiments, especially highlighting the beneficial role in minimizing the formation of gas bubbles at the CE and improving convective mass transport in the by‐pass channel.

However, in order to get a complete picture of the liquid cell behavior in practical conditions for EC‐LPTEM experiments, a better understanding of the gas bubble formation at the WE area must be obtained. To this aim, in situ experiments were carried out inside the transmission electron microscope, using the transmitted electron intensity to precisely track the presence of gaseous products at the WE during electrochemical stimulation of the liquid cell. As shown in Figure S1 (Supporting Information), when the viewing area is filled with gas (Figure S1, Supporting Information right), the transmitted electron intensity rises, due to the lower scattering power of gas compared to liquid. In this way, it is possible to correlate the presence of gas at the WE region with specific electrochemical conditions as a function of time. For the in situ experiments, large silicon chips with glassy carbon working electrodes were selected, since glassy carbon is inert and stable in a large potential range and it also generates lower background contrast in images compared to platinum.^[^ ^37^, ^38^ ^]^

Concerning the electrochemical stimulation, the experimental protocol mirrored the one discussed for the ex situ experiments. Also for this case, different flow rates (300, 1200, and 2500 µL h^−1^) of the CO_2_‐saturated 0.1 M KHCO_3_ electrolyte were investigated.

During each experiment, the flow rate, the electron beam energy and the primary beam intensity were kept constant. Therefore, the variations in intensity are exclusively related to the production of the gaseous products induced by changes of the electrochemical state of the system (see Movie S3, Supporting Information).

**Figure 3:**
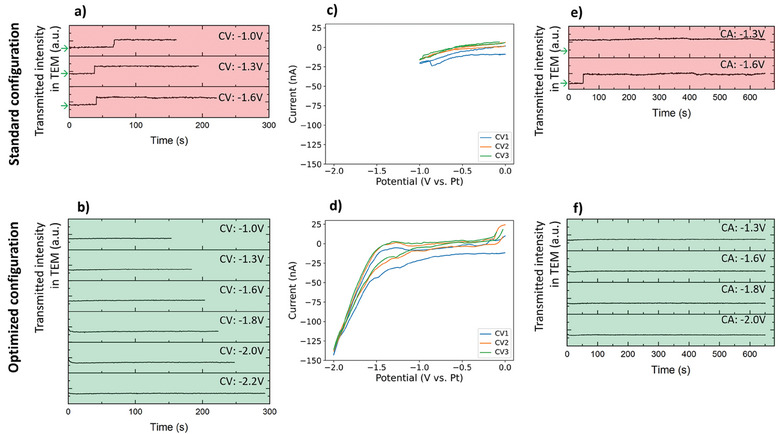
Transmitted electron intensity curves obtained at WE, acquired during cyclic voltammetry cycles for a) standard and b) optimized cell. Representative cyclic voltammetry cycles for c) standard and d) optimized cell. Transmitted electron intensity curves obtained at WE, acquired during chronoamperometry for e) standard and f) optimized cell. Color code: GREEN – cell fully filled with liquid, RED – cell saturated with gas. The green arrows indicate the transmitted electron intensity corresponding to the cell fully filled with liquid. The selected flow rate was 1200 µL h^−1^.

**Figure** **3** provides an overview of the transmitted electron intensity curves acquired during CV and CA experiments at 1200 µL h^−1^ flow rate. Abrupt changes in the transmitted electron intensity followed by a plateau are directly related to a condition where the electrolyte layer is very thin or even completely absent. Based on the results provided in Figure 3, the difference between the two different flow configurations is dramatic. In the standard configuration, gaseous products completely saturate the viewing window when −1 V versus Pt potential is reached through CV. This is repeatable and confirmed with several CV experiments with increasing potential window. It is interesting to notice that this phenomenon correlates with the ex situ experiments, although they were conducted with platinum WEs, which in principle should enhance the production of H_2_ through HER. These finding suggest that the main role in the gas saturation of the cell at negative potentials versus Pt may not be played by the WE, at least in the standard configuration, but by the CE. Based on these results, it is clear that with the standard configuration it is not possible to carry out CV or CA experiments at potentials < −1 V versus Pt (≈ – 0.2 V versus RHE) with the WE region fully filled with liquid. It is important to add that it was possible to run CA at −1.3 V and −1.6 V with a partially filled liquid cell and there was a current flow between WE and CE, possibly due to residual thin electrolyte layer. However, it is not ideal to carry out the experiments in these conditions, since the exact influence of large gas bubbles on the electrochemical behavior of the cell is still not well understood. It is, however, clear that the presence of large gas bubbles on the WE region leads to reduced area of contact between the electrode and electrolyte. This can influence or limit the supply of the fresh electrolyte and removal of the produced species during the catalytic activity. Concerning the recovery time, after each experiment the fresh electrolyte was flowed in the cell for at least 10 min or longer to assure that the cell was refilled again. The OCP was monitored within this time and only when this was stable, and the intensity was back to the initial conditions (filled cell) the following experiment was run. A similar unstable behavior was confirmed for the other studied electrolyte flow rates (see Figure S2, Supporting Information for 300 and 2500 µL h^−1^). At the flow rate of 300 µL h^−1^ some bubbles were created already during the cyclic voltammetry in the range 0 to −1.0 V versus Pt. When the electrolyte was flowed at the rate of 2500 µL h^−1^, it was possible to perform the electrochemical stimulation in the range 0 to −1.0 V versus Pt without generation of bubbles and/or dewetting of the electrode. This is an indication that enhanced flow rates may have beneficial effects for increasing the range of accessible potentials. However, higher flow rates may result in higher pressure experienced by the electron‐transparent Si_3_N_4_ membranes, which could possibly lead to mechanical failure and subsequent liquid leakage in the microscope column.

With the *diffusion* cell configuration, the range of experimental conditions compatible with the filled cell is greatly enlarged. Specifically, at the flow rate of 1200 µL h^−1^, it is possible to access potential values down to −2.0 V versus Pt with both CV and CA experiments, reaching conditions which are significant for energy applications. This is reflected also by the comparable behavior of multiple successive CV cycles (see Figure 3d). Similar stable behavior was observed for the other studied electrolyte flow rates (see Figure S3, Supporting Information for 300 and 2500 µL h^−1^). At the flow rate of 300 µL h^−1^ it is possible to perform the electrochemical stimulation without production of the bubbles in a very large potential window. Gas bubbles are created only during the cyclic voltammetry in the range 0 to −2.0 V versus Pt. When the flow rate is increased to 2500 µL h^−1^ it is possible to access potential values down to −2.0 V with both CV and CA experiments, similarly to the 1200 µL h^−1^ flow rate. The formation of large bubbles starts during the cyclic voltammetry in the range 0 to −2.2 V versus Pt. It is worth noting that the accessible potentials values are more negative compared to the ex situ experiments. This may be a consequence of the different WE electrode material, since glassy carbon is less active toward HER in comparison to platinum. However, as previously mentioned, the WE material did not play a huge role in the standard flow configuration, concerning the accessible electrochemical conditions. Another possible explanation may be related with the bulging of the Si_3_N_4_ membranes. It is well‐known that the electron‐transparent Si_3_N_4_ membranes experience bulging^[^ ^39^ ^]^ while the liquid cell is placed in the high‐vacuum of the electron microscope column, thereby increasing the available volume for the liquid electrolyte (see Figure S1, Supporting Information). As a consequence of a larger volume of the liquid electrolyte directly over the WE region, an increased production of gas (i.e., more negative potentials) is required at the WE to reach the solubility limit for the formation of bubbles.

As shown in Figure 3f and Figure S3 (Supporting Information), the improved flow configuration allows to carry out chronoamperometry measurements for prolonged times and at potentials which were previously not accessible with the standard flow configuration (see Figure S4, Supporting Information for CA carried out at −2.0 V versus Pt). It must be stressed that chronoamperometric techniques are widely exploited for the evaluation of the catalyst performance in reactions relevant for the energy transition. Thus, the optimized *diffusion* cell design paves the way for future EC‐LPTEM studies, shedding light on the morphological and/or structural evolution of the catalyst and the influence of these changes on activity, selectivity, and stability. As a final remark it is important to note that even with the *diffusion* cell configuration it is possible to enhance the resolution of the microscopic analysis by saturating the liquid cell with gas, which has lower scattering power compared to the liquid electrolyte. For all tested flow rates, at potentials more negative than −2.2 V the gas production is overwhelming and the cell is saturated with gas. Although a limitation in the available potential window, this phenomenon provides the opportunity to selectively decrease the electrolyte thickness, allowing for the extraction of structural information from images and diffraction patterns. Thanks to the optimized liquid flow configuration, fast recovery of the “filled cell” condition is obtained within 1–2 min once the in situ structural analysis is concluded.

### 2.3 Zn Electrodeposition

Ex situ and in situ comparative flow experiments showed that the optimized geometry allows to perform electrochemical experiments in conditions which were not accessible with the standard flow configuration. Direct proof of these positive effects requires an electrochemical experiment that would not be possible using a standard flow configuration with the same TEM holder.

Among the possible EC‐LPTEM experiments, electrodeposition processes are ideal candidates for proving the beneficial effects of the modified geometry. This is because electrodeposition in the “thin electrolyte” condition would result in a very limited material growth, since the chemical species involved in the process would immediately be depleted. Moreover, electrodeposition processes attract much attention since they are employed in the manufacturing industry as fabrication techniques. They are also of great importance to unveil the electrochemical mechanisms involved in rechargeable batteries during charge‐discharge cycling.^[^ ^40^ ^]^ In this context, various experiments have already been performed with commercial chips, thanks to the fact that they did not require an aqueous environment (i.e., traditional Li‐ion batteries).^[^ ^41^, ^42^ ^]^ However, due to the need for more sustainable devices, researchers are also focusing on batteries based on different chemical elements (Zn, Mg, etc.), thus demanding water‐based electrolytes^.[^ ^43^ ^]^

For the previously mentioned reasons, we chose the electrodeposition of Zn as a proof‐of‐concept, showing the experimental implications of adopting the two different liquid cell geometries. The electrocatalytic setup in EC‐LPTEM differs from real‐world battery scenarios, where electrodes typically have larger surface areas and higher electrolyte concentrations. In the miniaturized cell, the conditions must be optimized to study the process effectively. However, due to the smaller size and thickness of the electrodes and the extremely small liquid volume, electrolyte transport phenomena, including ion mobility, are significantly impacted.^[^ ^44^ ^]^ Since the analysis of the implications in the charge‐discharge process of Zn‐air batteries is out of the scope of this work, we just focused on the application of the −0.5 µA cathodic current to show the electrodeposition process. Due to the significant water splitting occurring at the Pt WE and CE during the electrodeposition process, it is not possible to perform the experiment with the standard commercial set‐up since the liquid cell is immediately filled with the gaseous products (see Movie S4, Supporting Information). This has already been reported with a comparable liquid cell geometry by Sasaki et al.^[^ ^45^ ^]^ Production of gas bubbles (at the WE) during in situ Zn deposition experiments has also been reported with other convection‐governed liquid cell geometries.^[^ ^46^ ^]^ As a possible solution to the problem, Sasaki and coworkers minimized O_2_ production at the CE through the ex situ deposition of Zn on the Pt CE, therefore successfully obtaining the Zn deposition through adding an extra technological step to the experiment. With the *diffusion* cell geometry, it is possible to observe the Zn electrodeposition without any specific preparation step.

**Figure 4:**
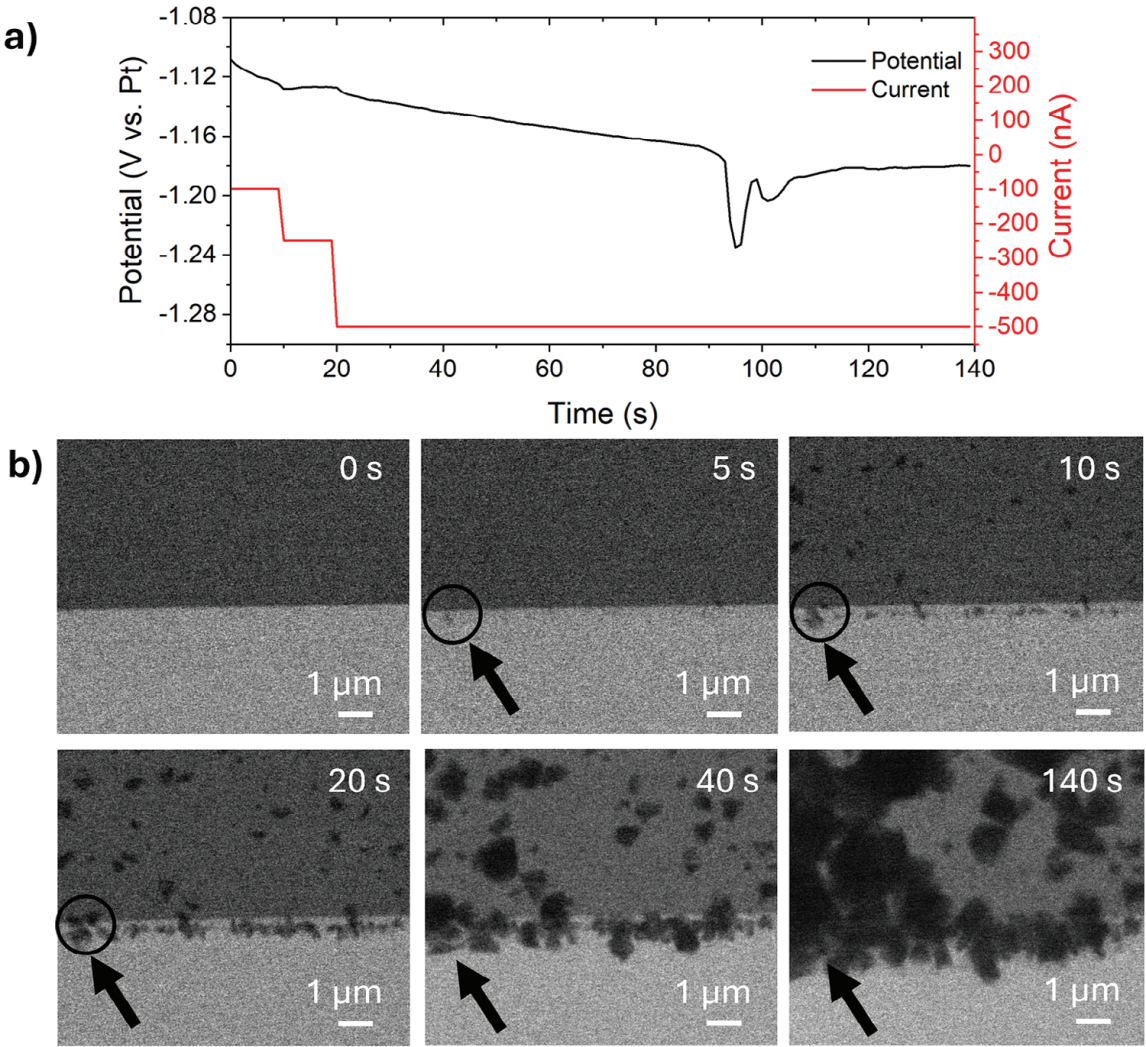
Electrodeposition of Zn on the Pt WE at −0.5 µA: a) chronopotentiogram and current profile applied during the electrodeposition process, and b) BF‐STEM images at indicated times with the same magnification. The electrolyte flow rate was 1200 µL h^−1^.

The applied current over time is shown in **Figure** **4**a: two intermediate steps with increasing current (−0.1 µA, −0.25 µA) were implemented for 10 s each, before reaching the required −0.5 µA current for the galvanostatic electrodeposition process, lasting 120 s. Throughout the entire process, the potential between the WE and the Pt RE is more negative than −1.1 V versus Pt (see Figure 4a), a condition which is critical for the standard flow geometry, that, as previously discussed, causes complete gas saturation of the cell (see Movie S4, Supporting Information). Figure 4b shows significant images extracted from the Movie S5 provided in the Supporting Information. It is interesting to note that electrodeposition already starts at the initial intermediate steps: −0.1 µA from 0 to 10 s, −0.25 µA from 10 to 20 s. Based on the image acquired at 5 s, it is possible to detect the presence of nucleating Zn nanostructures, which grow bigger in the following 15 s. Once the target −0.5 µA current is established, more particles are generated and those already present grow in size. The movie shows that it is possible to track the morphological evolution of the electrodeposited nanostructures while the viewing area is filled with the electrolyte all the time. The measured potential at which electrodeposition occurred during the galvanostatic deposition shown in our study (Figure 4a) is close to the estimated potential of Zn^2^⁺/Zn electrode pair versus the Pt reference electrode (see the “Experimental Methods” section).

It is important to add that also in *diffusion* cell geometry it is possible to create gas bubbles to reduce the liquid to a thin film that remains covering the windows, the electrodes, and the sample. This permits to obtain resolved structural and chemical analyses at the nanoscale in liquid conditions, in similar way as with the standard chip, however in the new configuration this is performed on demand at relatively negative potentials.

BF‐STEM images (see Figure S7, Supporting Information) show the electrodeposited nanostructures observed when the cell was brought to the thin film condition by providing electrochemical stimulus through CV in the potential range of 0 to −2.0 V versus Pt. As can be seen, the visibility is drastically improved, and very thin platelets are clearly observable in this condition, which was not achievable when a cell with 650 nm spacer is fully filled. The electrolyte is quickly recovered when the water‐splitting voltage is no longer being applied. The deposition was characterized by post in situ field emission scanning electron microscopy (FESEM) analysis to assess the actual presence of Zn on the WE. FESEM images (see Figure S5, Supporting Information) confirm the deposition of a Zn‐containing layer in the form of nanostructured foils. Based on energy dispersive X‐ray spectroscopy (EDS) maps (Figure S5, Supporting Information), the WE presents a uniform coverage of Zn and O atoms, which can be related to the presence of ZnO. Due to the high tendency of metallic Zn to oxidize after air exposure, it is reasonable to consider that during the application of current, only metallic zinc was deposited, as already supposed by Sasaki et al.,^[^ ^45^, ^47^ ^]^ with subsequent air‐induced oxidation while transferring the chip from the liquid cell to the FESEM chamber. It is also important to note that the Zn electrodeposition process was completely selective toward the WE, as evidenced by EDS analysis performed at the CE and RE, which does not show any signal related to Zn (Figure S6, Supporting Information).

This experiment demonstrates the efficacy of the optimized configuration in speeding up the removal of gaseous products due to water splitting reaction. This is a feature of paramount relevance when considering in situ */ operando* experiments conducted in aqueous environment, as gaseous side products cannot be avoided in most electrochemically relevant conditions.

### 2.4 CO2RR Experiment with Cu‐Based Catalyst

**Figure 5:**
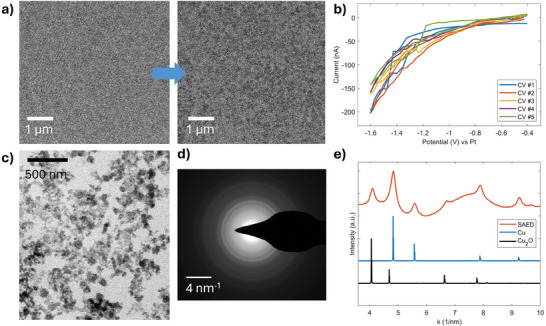
EC‐LPTEM study of Cu‐based catalyst under CO2RR operating conditions: representative BF‐STEM images a) of the Cu‐based catalyst showing the morphological evolution during CV b). Liquid cell partially filled with gas, acquisition of: BF‐STEM image c), SAED pattern d) with corresponding rotationally‐averaged, background‐subtracted pattern e). The electrolyte flow rate was 1200 µL h^−1^.

The implications of the *diffusion* cell geometry for CO2RR experiments were also investigated in the presence of a catalyst material. A Cu‐based catalyst was deposited by sputtering and confined in the WE region with a shadow mask. In this way, coverage of the WE region with the catalyst material is optimized (see Figure S8, Supporting Information). The thin film is constituted of nanostructured Cu crystalline grains and is expected to partially oxidize after being exposed to the 0.1 M KHCO_3_ solution at OCP conditions.^[^ ^48^ ^]^ As shown in **Figure** **5**a, the morphological evolution of the catalyst was observed with cyclic voltammetry stimulation at cathodic potentials. Relevant conditions for CO2RR were reached (−1.6 V vs. Pt ≈ −0.8 V versus RHE), where the oxidized Cu is expected to experience reduction and reach an active state for CO2RR.^[^ ^49^ ^]^ It is worth noticing that successive CV cycles (see Figure 5b) do not show drastic changes in the measured current since the cell is fully filled with liquid, thanks to mass transport conditions dictated by the *diffusion* cell geometry. STEM images provide an indication of the morphological changes of the thin film, suggesting possible dissolution/crystallization or crystal aggregation phenomena. The morphology gradually changes from a compact thin film to sparse islands of copper‐based material (Figure 5a). By enlarging the potential window to −2.0 V versus Pt, is it possible to reach an intermediate condition where the cell is partially filled with gas (see Movie S6, Supporting Information), improving the resolution of the imaging process (Figure 5c) and allowing for the acquisition of in situ selected area electron diffraction (SAED) patterns (Figure 5d). Investigation by SAED confirms the co‐presence of Cu and Cu_2_O crystalline phases (Figure 5e), providing in situ information of structural changes experienced by the catalyst inside the liquid cell. Although a complete investigation of the degradation pathway of the Cu‐based catalyst is out of the scope of this work, this proof‐of‐concept experiment shows important results: i) consistent electrochemical stimulation is applied in conditions relevant for CO2RR for a prolonged period of time with the cell fully filled with liquid, ii) it is possible to enhance the resolution by applying more negative potentials (−2.0 V vs. Pt ≈ −1.2 V vs. RHE), to obtain structural information.

## 3 Conclusion

The role of cell design and liquid flow configuration in EC‐LPTEM was investigated by means of two different concepts: the conventional cell with planar geometry and the *diffusion* cell with on‐chip micrometric by‐pass channel. While with the standard geometry the three electrodes (WE, RE, CE) all lie in a sub‐µm thick nanochannel, in the optimized geometry the CE and RE are in a 10 µm – thick bypass channel, therefore being exposed to a larger electrolyte volume. Through ex situ comparative flow experiments with optically‐transparent large glass chips, it was shown that the formation of gas bubbles at the CE is minimized in the *diffusion cell* geometry because the CE is exposed to a larger volume of electrolyte and the gaseous products are more efficiently brought to the outlet by the optimized liquid flow configuration. In situ comparative experiments at cathodic potentials in the electron microscope confirm that the *diffusion cell* geometry shifts the congestion of the viewing area (corresponding to the WE) with gaseous products to more negative potentials. It is therefore possible to enlarge the potential window for the above discussed in situ electrochemical experiments to ≈ −2.0 V versus Pt (≈ −1.2 V vs. RHE), while keeping the liquid cell fully filled with electrolyte. It is important to notice that it is also possible to momentarily enhance the resolution of the TEM analysis by intentionally saturating the liquid cell with gas by applying potentials more negative than ≈ −2.0 V versus Pt (≈ −1.2 V vs. RHE), thus establishing, when needed, the “thin electrolyte” condition.

Although the comparative experiments were carried out only at cathodic potentials, it is expected that the beneficial effects of the *diffusion cell* geometry also apply to anodic potentials. The same experimental approach may be employed to understand the specific conditions for the operation of the liquid cell fully filled with electrolyte. In this regard, it is also worth stressing the importance of the optically transparent large glass chips for providing a preliminary investigation of the response of the whole cell to the electrochemical stimulation.

It was demonstrated that recently introduced designs^[^ ^33^ ^]^ pave the way for future in situ / *operando* experiments in electrochemical conditions which are more directly comparable with the macroscopic counterparts in the context of heterogeneous catalysis for energy transition. The possibility of studying the dynamical evolution of the morphology and structure of the catalysts over a wider range of electrochemical conditions is expected to provide insight into the fundamental mechanisms that influence their activity, selectivity and stability.

The expected benefits also apply to processes of interest for the energy storage research field, such as electrodeposition.

As a final remark, this work shows the critical role of cell geometry and liquid flow configuration in enabling EC‐LPTEM experiments at electrochemical conditions of interest for the energy transition. It is envisioned that further optimization of the flow configuration and of other key elements in the liquid cell (for example the geometry of the electrodes and their relative positions), will significantly impact the experimental control and data interpretation.

## 4 Experimental Section

### Experimental Set‐Up

The influence of the liquid flow configuration during EC‐LPTEM experiments is investigated by comparing two different microfluidic cell geometries: the *standard* cell and the *diffusion* cell. The two concepts were experimentally implemented in the *Poseidon Select* holder (Protochips Inc.). The miniaturized liquid cell was delimited by two chips (see Figure 1): the large chip provides electrochemical functionality with a MEMS‐based approach, while different designs of the small chip provide control on the liquid cell geometry and therefore on the liquid flow configuration. The thickness of the liquid cell was determined by the spacers placed on the large as well as on the small chips and the liquid cell was sealed using a gasket.

In this work, we compare two different flow configurations, obtained by two different designs of the small chip:^[^ ^33^ ^]^

i) the standard configuration (see Figure 1 top left), Protochips commercial bottom e‐chips: EPB‐55DNF, 550 µm x 50 µm viewing window (SiN_x_, 50 nm thickness), 150 nm spacer height.
ii) the diffusion cell configuration (see Figure 1 bottom left), Protochips commercial bottom e‐chips: ECB‐55DNF‐FM, 550 µm x 50 µm viewing window (SiN_x_, 50 nm thickness), 150 nm spacer height.

The diffusion cell design results in dramatically reduced diffusion length in the proximity of the viewing area, while simultaneously confining convective mass transport outside the viewing area. The diffusion cell concept was obtained by introducing an on‐chip by‐pass channel in the small chip, while keeping the electron‐transparent window on an elevated “island” which confines the nanochannel only to the imaging area. After optimization of the geometrical parameters based on numerical models and specific technological constraints, the physical implementation of the diffusion cell concept has the following features: i) on‐chip by‐pass channel height: 10 µm, formed between four pentagonal plateau, ii) the central island extends over ≈ 120 × 600 µm^2^, iii) spacers, which define the thickness of the liquid were located at the corners of four pentagonal plateau.^[^ ^33^ ^]^

Concerning the large chips, they provide the electrochemical functionality in a three‐electrode configuration, with a linear WE in the middle with a semi‐circular CE around it and a square‐shaped RE on its bottom right. The RE and CE were made of platinum, while for the WE there was the possibility of choosing between platinum or glassy carbon. All the Pt electrodes were ≈ 75 nm thick, while the glassy carbon electrode was 60 nm thick. Commercial silicon large chips ECT‐45PT and ECT‐45CR were used (Pt and glassy carbon WE respectively), with viewing window 550 µm x 40 µm (SiN_x_, 50 nm thick). In this work, *ad‐hoc* large chips made of glass (from Protochips) were also used to carry out experiments under the optical microscope (referred to in the text as ex situ experiments). The optical transparency of the glass large chips and the use of a stereomicroscope allows the observation of RE, CE and WE at the same time. These chips feature three Pt electrodes with the same geometry as the commercial standard silicon large chips. The spacer on all types of the large chips was 500 nm. All the chips (standard and modified) are manufactured by Protochips, Inc. The electrochemical stimulation is provided with a Gamry Reference 600+ potentiostat. In all experiments, the potential of the platinum pseudo‐reference electrode is ≈ 0.8 V versus the reversible hydrogen electrode (RHE), as previously reported in the literature^[^ ^48^, ^50^ ^]^ therefore V versus Pt ∼ V versus RHE − 0.8 V. The flow rate during the experiments was controlled with a Harvard Apparatus 11 Elite syringe pump.

### Comparative Flow Experiments

A carefully designed experimental protocol had been implemented for the comparison between the two liquid cell set‐ups, which was targeted toward electrocatalytic CO_2_ reduction reaction applications in aqueous electrolyte. Specifically, CO_2_‐saturated 0.1 M KHCO_3_ was selected as aqueous electrolyte, since it is the most common choice for this application.^[^ ^51^, ^52^ ^]^ In fact, it was able to collect and dissolve CO_2_, which was in equilibrium with HCO_3_^−^ species, and it has good buffering capability, keeping the pH around neutral values.^[^ ^53^ ^]^ Electrochemical techniques such as Cyclic Voltammetry (CV) and Chrono‐Amperometry (CA) were employed for the evaluation of the influence of liquid flow configuration on the liquid cell behavior. Different flow rates were examined: 300, 1200, and 2500 µL h^−1^.

Two different classes of experiments were carried out: ex situ experiments under the optical microscope, and in situ experiments in the transmission electron microscope. For ex situ experiments, large chips made of glass were used since they provide optical transparency. For in situ experiments, standard large silicon chips with a glassy carbon working electrode were used since glassy carbon is commonly used as current collector for CO_2_RR^[^ ^51^ ^]^ and it has lower activity toward HER compared to Pt.^[^ ^54^ ^]^ Successive cyclic voltammetry measurements were performed with increasing potential window at 50 mV s^−1^ scan‐rate, in order both to condition the electrochemical cell and to test exactly at which potential the gas production begins to be unbearable for the chosen flow rate. If the CVs did not produce a huge amount of gas at certain potentials (−1.3, −1.6, −1.8, −2.0 V vs. Pt) then, also 10 min chronoamperometry was performed to preliminary understand if the cell could sustain the potentials of interest for an extended period of time. Before the CA, a potential sweep was employed to pass from the Open Circuit Potential (OCP) to the target value for the CA with 50 mV s^−1^ scan‐rate. The behavior of the cell was investigated during the electrochemical stimulation by means of the optical microscope (ex situ experiment) or the TEM (in situ experiment). By exploiting these techniques, it was possible to correlate the electrochemical response of the cell with the evolution of the formation of the gaseous products.

During the electrochemical stimulation under the electron beam, the formation of gas bubbles was monitored by using a method developed in a previous work.^[^ ^31^ ^]^ Briefly, variations in the transmitted electron intensity in parallel beam illumination were tracked over time. When the presence of gas bubbles causes dewetting of the region under study, the transmitted electron intensity rises due to the lower scattering power of the gas phase with respect to the electrolyte.

The electron‐optical parameters (excitation of all the TEM lenses) are kept the same throughout all the experiments. The in situ experiments were carried out in a Tecnai F20ST transmission electron microscope (Thermofisher, former FEI), operating in TEM mode at 200 kV accelerating voltage. The illumination conditions were kept the same through all the in situ experiments, with a ≈ 8 e^−^/nm^2^/ dose‐rate.

### Zn Electrodepositon

Electrodeposition of Zn nanostructures in aqueous electrolyte was carried out using 0.1 M ZnSO_4_ in ultrapure water, with a flow rate of 1200 µL h^−1^. For this experiment the cell was mounted by means of a silicon large chip with Pt electrodes (500 nm spacer), and a diffusion cell small chip with a 150 nm thick spacer. The deposition was carried out by chronopotentiometry (CP), where the target current of −0.5 µA was reached through two steps with increasing current values (−0.1 and −0.2 µA) for 10 s each. The target current was, then, kept fixed for 120 s to follow the growth mechanism of Zn dendrites. The measured potential values during the galvanostatic deposition process are reported versus the platinum reference electrode. Considering the potential of the Zn^2+^ / Zn couple of −0.76 V versus Standard Hydrogen Electrode (SHE),^[^ ^55^ ^]^ the potential versus RHE may be calculated as follows: V versus RHE = V versus SHE + 0.059*pH = – 0.346 V for pH = 7.

Using the relation V versus Pt ∼ V versus RHE − 0.8 V we obtain ≈ −1.15 V versus Pt potential for the Zn^2+^ / Zn couple.

The electrodeposition procedure follows the approach reported by Sasaki et al.,^[^ ^45^ ^]^ except for the liquid flow rate (1200 µL h^−1^) and for the deposition time interval, which was much longer in this work. In situ TEM imaging of the electrodeposition process had been carried out with a Tecnai F20ST microscope (Thermofisher, former FEI), operated at 200 kV accelerating voltage, in STEM mode (dose per frame ≈ 1e^−^/nm^2^). Post in situ Field Emission Scanning Electron Microscopy (FESEM) characterization of the electrodeposited nanostructures was performed with dual‐beam FIB‐SEM workstation (Auriga model by Zeiss) equipped with a Silicon Drift Detector (Oxford Instruments) for Energy Dispersive X‐ray spectroscopy (EDS).

### CO2RR Experiment with Cu‐Based Catalyst

The morphological characterization of Cu‐based catalyst in conditions relevant for CO2RR was performed in CO_2_‐saturated 0.1 M KHCO_3_ electrolyte, with 1200 µL h^−1^ flow rate. The cell was constituted of a silicon large chip (WE: glassy Carbon, CE: Pt, RE: Pt; 500 nm spacer) and a diffusion cell small chip (150 nm spacer). A thin Cu film was deposited by RF magnetron sputtering (Quorum Technologies Ltd Q150T) coupled with a mask with optimized conditions (50 mA, 130 s) to obtain an electron‐transparent film on the WE region (see Supporting Information). A series of CV experiments was carried out at cathodic potentials, with 50 mV s^−1^ scan‐rate. In situ STEM images were acquired with a Tecnai F20ST microscope (Thermofisher, former FEI), operated at 200 kV accelerating voltage (dose per frame ≈ 1e^−^/nm^2^).

## Conflict of Interest

The authors declare no conflict of interest.

## Supporting information



Supporting Information

Supplemental Movie 1

Supplemental Movie 2

Supplemental Movie 3

Supplemental Movie 4

Supplemental Movie 5

Supplemental Movie 6

## Data Availability

The data that support the findings of this study are available from the corresponding author upon reasonable request.
