# Supplementary material for: Electrochemical Liquid Phase TEM in Aqueous Electrolytes for Energy Applications: the Role of Liquid Flow Configuration
Source: Small Methods. 2024 Nov 27;9(3):2401718. doi: 10.1002/smtd.202401718 (PMC11926486; doi:10.1002/smtd.202401718)
Supplement: Supplementary file 1 — Supporting Information [file SMTD-9-2401718-s001.pdf]

small
methods

Supporting Information

for *Small Methods*, DOI 10.1002/smtd.202401718

Electrochemical Liquid Phase TEM in Aqueous Electrolytes for Energy Applications: the Role of Liquid Flow Configuration

Katarzyna Bejtka, Marco Fontana, Cecilia Irene Gho, Stefan Merkens, Andrey Chuvilin, Candido Fabrizio Pirri and Angelica Chiodoni*

# – Supporting Information –

Electrochemical liquid phase TEM in aqueous electrolytes for energy applications: the role of liquid flow configuration

Katarzyna Bejtka[a,b,]†, *Marco Fontana*[a,b,]†[*], *Cecilia Irene Gho*[a,b], *Stefan Merkens*[c], *Andrey Chuvilin*[c,d], *Candido Fabrizio Pirri*[a,b], *Angelica Chiodoni*[b]

[a] Department of Applied Science and Technology, Politecnico di Torino, Corso Duca degli Abruzzi 24, 10129 Torino, Italy

[b] Center for Sustainable Future Technologies @Polito, Istituto Italiano di Tecnologia, Via Livorno 60, 10144 Torino, Italy

[c] Electron Microscopy Laboratory, CIC nanoGUNE BRTA, Tolosa Hiribidea 76, Donostia, San Sebastian 20018, Spain

[d] Ikerbasque, Basque Foundation for Science, 48013 Bilbao, Spain

† Authos contributed equally to this work.

(* corresponding author: marco.fontana@polito.it)

Contents

1. Supplementary Figures regarding In-situ 2
2. Supplementary Figures regarding Zn electrodeposition 4
3. Supplementary Figures regarding Cu-catalyst characterization 7
4. Supplementary note regarding convection diffusion model of solute removal 8
5. Supplementary Movies: list and details 9

1. Supplementary Figures regarding In-situ experiments

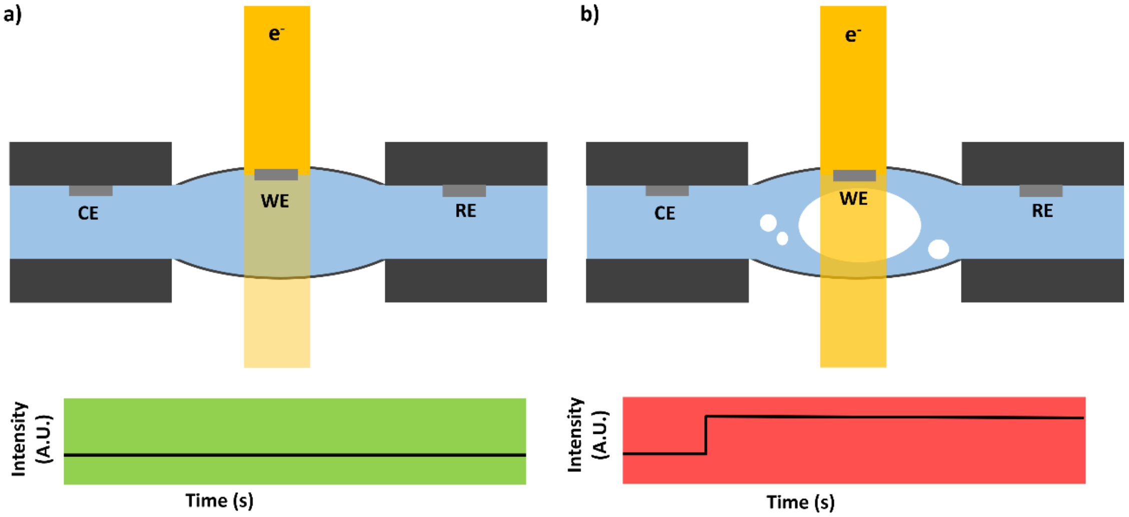

Supplementary Figure S1: schematics in cross-sectional view of the time-tracking of transmitted electron intensity to discriminate between: (a) the WE region fully filled with liquid, (b) the WE region with gas bubbles.

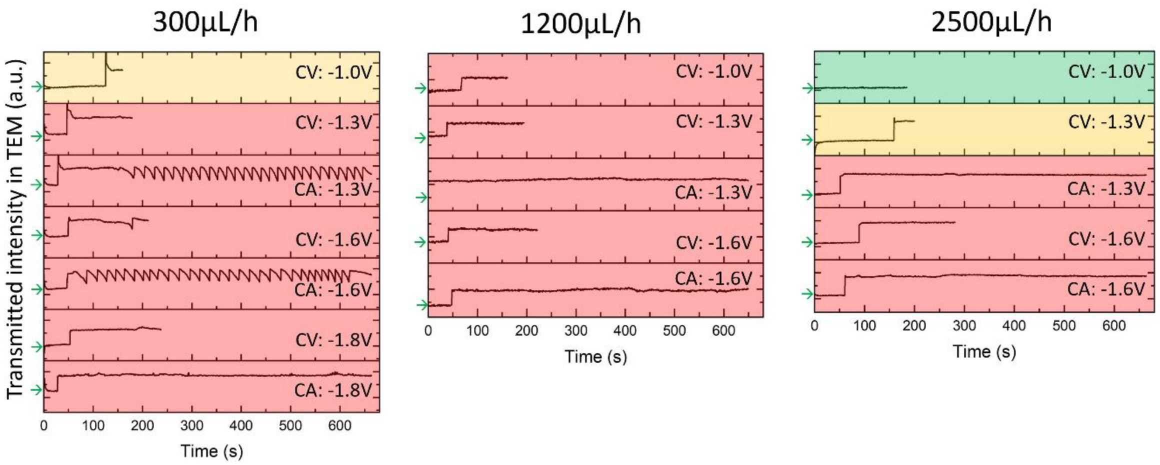

Supplementary Figure S2: transmitted intensity curves at the WE acquired during electrochemical stimulation for the standard cell. The flow was 300 µL/h, 1200 µL/h and 2500 µL/h as indicated in the figure. Color code: GREEN - cell fully filled with liquid, RED - cell saturated with gas (once the most negative potential is reached or before), and YELLOW – the cell saturates with gas after it has been full for more than one cycle of the CV. The green arrows indicate the transmitted electron intensity corresponding to the cell fully filled with liquid.

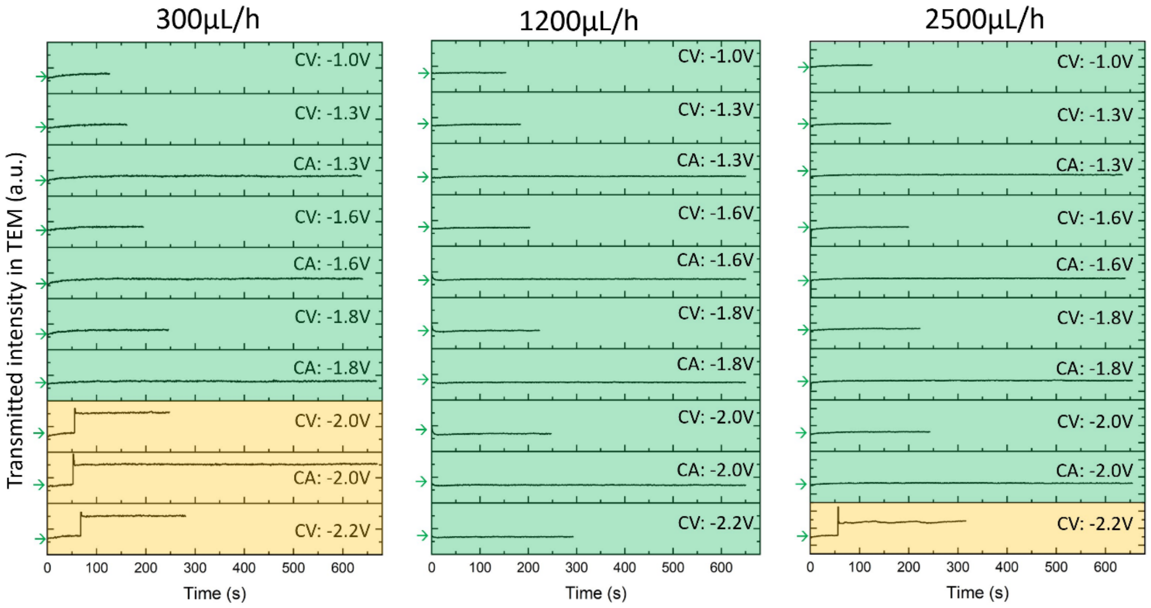

Supplementary Figure S3: transmitted intensity curves at the WE acquired during the electrochemical stimulation for the optimized cell geometry. The flow was 300 µL/h, 1200 µL/h and 2500 µL/h as indicated in the figure. Color code: GREEN - cell fully filled with liquid, RED - cell saturated with gas (once the most negative potential is reached or before), and YELLOW – the cell saturates with gas after it has been full for more than one cycle of the CV. The green arrows indicate the transmitted electron intensity corresponding to the cell fully filled with liquid.

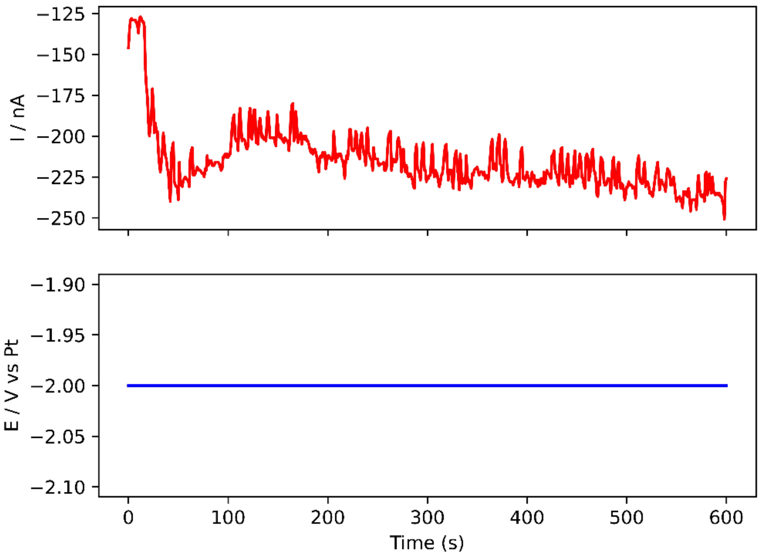

Supplementary Figure S4: in situ chronoamperometry carried out in CO2-saturated 0.1M $KHCO_3$ electrolyte at –2.0 V vs Pt, at 1200 µL/h flow rate with the optimized cell geometry.

2. Supplementary Figures regarding Zn electrodeposition

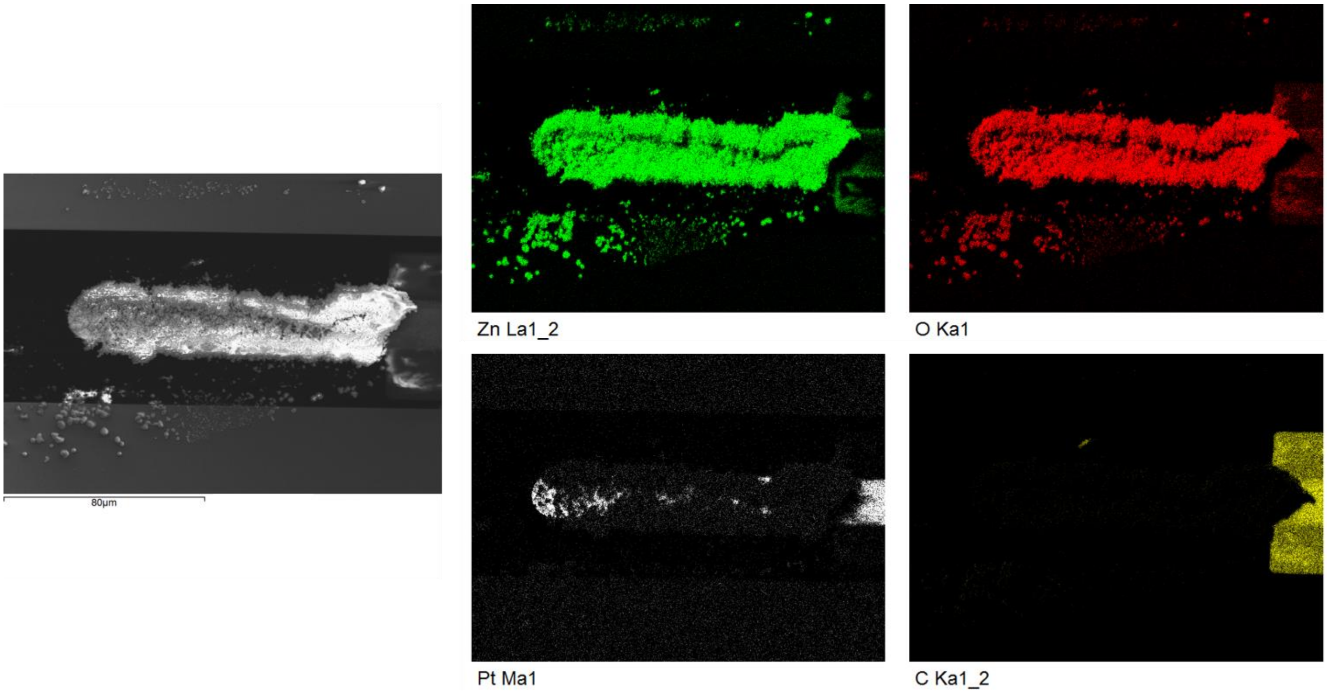

Supplementary Figure S5: FESEM image (left) and EDS maps (right) of the electrodeposited Zn on the WE acquired with a 8 keV electron beam. The Zn signal correlates with the oxygen due to the presence of the ZnO, resulting from the oxidation of the deposited metallic Zn due to air contact. It is still possible to detect Pt under Zn, underlining that the Zn layer is uniform but it has some differences in thickness on the electrode. The carbon signal is due to the polymeric resist.

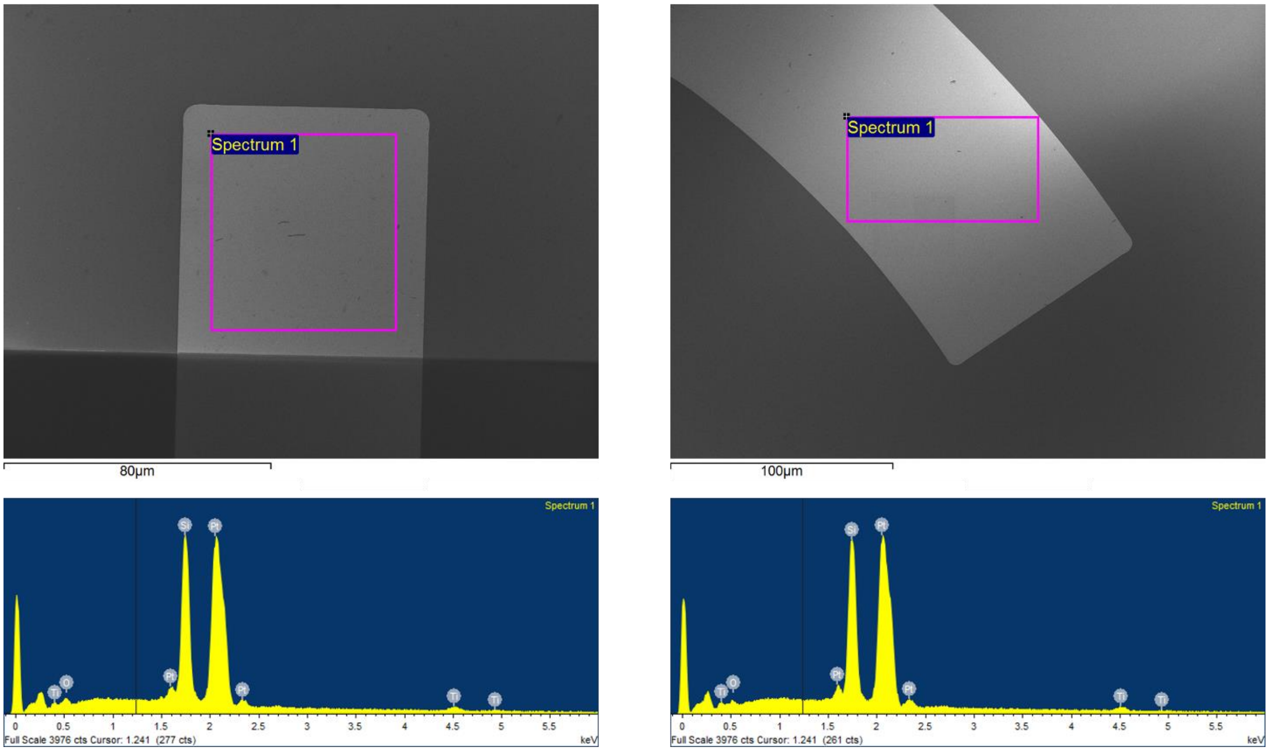

Supplementary Figure S6: FESEM images and EDS spectra of the RE (left) and CE (right), obtained with a 8 keV electron beam. Spectra show the presence of Pt and Ti, which are respectively the electrodes material and the metal used to improve its adhesion to the silicon nitride substrate. The presence of Zn is not revealed by EDS, thus confirming that the performed electrodeposition was selective towards the WE.

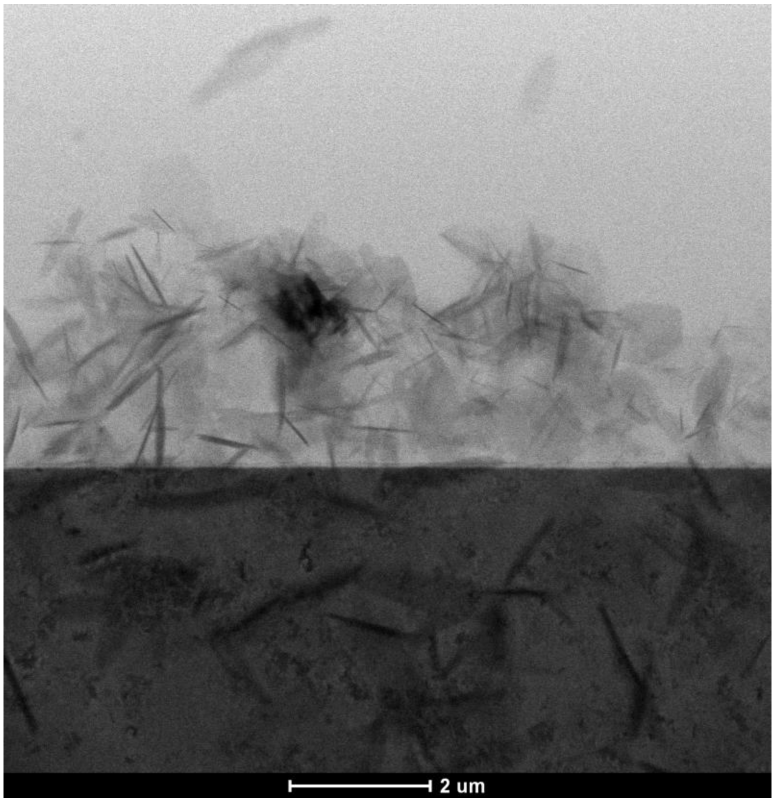

Supplementary Figure S7: BF-STEM image obtained from the Zn ED experiment once the cell was emptied.

3. Supplementary Figures regarding Cu-catalyst characterization

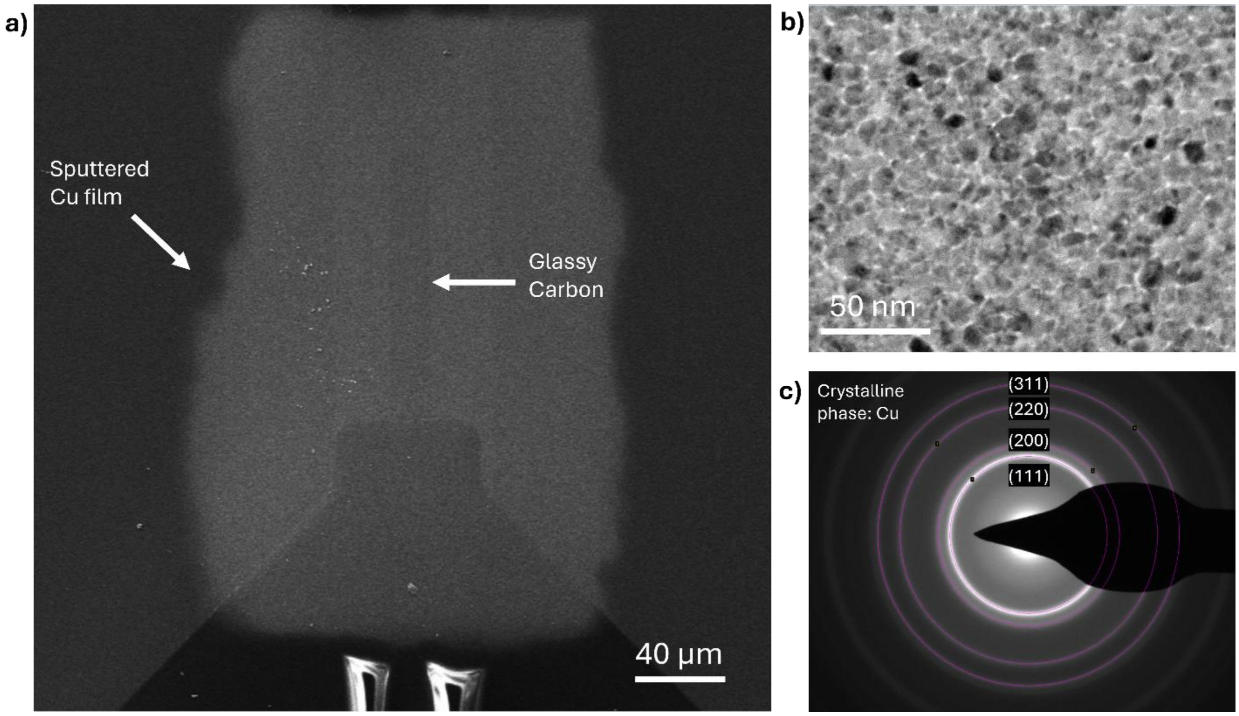

Supplementary Figure S8: FESEM image of the as-deposited Cu film on the large electrochemical silicon chip with glassy carbon electrode(a). BF-STEM image (b) and SAED pattern obtained using the Inspection holder by Protochips, which enables the analysis of the large chip in vacuum conditions.

4. Supplementary Note regarding convection diffusion model of solute removal

To estimate the electrochemical generation of species, previously established geometric models [1] were adjusted by implementing flux boundary conditions to a surface segment representative for the WE. The solute (with diffusion coefficient $D = 4.5 \times 10^{-9}$ m^2 s^{-1} to reflect H_2) was generated at surfaces already present in the underlying geometric models of the standard and optimized configuration, respectively. In both configurations, a quadratic surface in the center of the nanochannel was selected to reflect the WE. The gas generation rate (N_0 = 1 mmol m^{-2} s^{-1}) was estimated based on experimental current densities of 20 mA cm^{-2} (current: I = 500 nA, active area of the WE: A_{WE} = 2400 cm^2). H_2 was chosen as representative chemical species for the calculation since it is the most common product at the bare electrodes (especially Pt) at cathodic potentials considered in this work. For other gases which may be present in the viewing window, no drastic difference is expected, given that diffusion coefficient of all is in comparable order of magnitude [2], [3],[4].

The simulated steady state solute concentrations for both configurations are depicted in Supplementary Figure S9. Solute removal is confirmed to be dominated by convection in the standard configuration (Supplementary Figure S9 a), whereas radially symmetric concentration profiles in the optimized configuration indicate diffusion as dominant transport mechanism in the central nanochannel (Supplementary Figure S9 b). Noteworthy, the maximum solute concentration is reduced by ~25% in the optimized as compared to the standard configuration. Removal in the on-chip bypass channel is drastically improved due to enhanced convection leading to negligibly small solute concentration in proximity to the central nanochannel.

Despite the simplistic implementation, the models were proven useful to understand mechanisms governing solute removal in different flow reactor configurations and to semi-quantitatively interpret the experimental findings reported in the main manuscript. Advanced models that consider chemical (surface) reactions and gas bubble formation are expected to unleash additional insights.

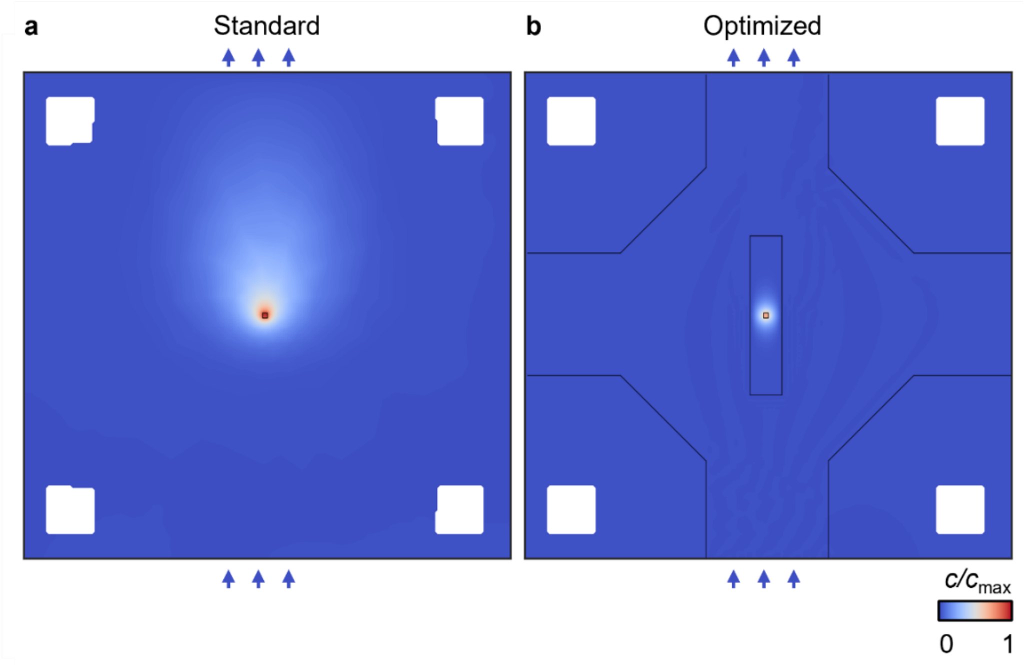

Supplementary Figure S9: Simulated steady state 2D concentration maps of a locally generated species in the standard (a) and optimized configuration (b). Blue arrows indicate direction of fluid flow. Small black squares in the center indicate the gas generation area (WE). In b, the surrounding rectangles represent the nanochannel; remaining black lines indicate channel walls of the on-chip bypass. The solute (with $D = 4.5 \times 10^{-9}$ m^2 s^{-1} to reflect H_2) is generated (rate: $N_0 = 1$ mmol m^{-2} s^{-1}) at the surfaces defined by the small black squares (20 × 20 µm^2 reflecting the WE; in a & b). Q_{total} was 3000 µL h^{-1}. The height of the nanochannel was $h_{NC} = 150$ nm. In the optimized configuration, the lateral extension of the central nanochannel was $w_{NC} \times l_{NC} = 120 \times 650$ µm^2 and the height of the on-chip bypass channel $h_{BP} = 10$ µm.

5. Supplementary Movies: list and details

Supplementary Movie 1

Description: Optical microscopy movie illustrating the electrochemical stimulation for the standard cell configuration, in detail during the CVs in the range -1.2 V to 0 vs Pt at 1200 µL/h electrolyte flow. The movie also shows the cell during 1 min after the CV was stopped.

The movie playback rate is ×4 times real time.

Supplementary Movie 2

Description: Optical microscopy movie illustrating the electrochemical stimulation for the diffusion cell configuration, in detail during the CVs in the range -1.8 V to 0 vs Pt at 1200 µL/h electrolyte flow. The movie also shows the cell during 1 min after the CV was stopped.

The movie playback rate is ×4 times real time.

Supplementary Movie 3

Description: Low magnification TEM movie illustrating the observation of the WE and transmitted intensity in TEM during the electrochemical stimulation for the standard cell configuration, in detail during the CVs in the range -1.8 V to 0 vs Pt at 300 µL/h electrolyte flow.

The movie playback rate is real time.

Supplementary Movie 4

Description: BF-STEM movie displaying the electrodeposition of Zn on the Pt WE in the standard cell configuration in 0.1 M $ZnSO_4$. The electrolyte was flowing at 1200 µL/h rate.

The movie playback rate is ×4 times real time.

Supplementary Movie 5

Description: BF-STEM movie displaying the electrodeposition of Zn on the Pt WE in the diffusion cell configuration in 0.1 M $ZnSO_4$ for 140 seconds. The electrolyte was flowing at 1200 µL/h rate.

The movie playback rate is ×4 times real time.

Supplementary Movie 6

Description: HAADF-STEM movie showing Cu-catalyst during the CO2RR experiment performed in the optimized cell configuration, in the intermediate condition where the cell is partially filled with gas. This condition was induced by provision of the electrochemical stimulation in the form of CVs in the range –2.0 V to 0 vs Pt. The electrolyte was flowing at 1200 µL/h rate.

The movie playback rate is ×2 times real time.

REFERENCES

[1] S. Merkens *et al.*, "Toward sub-second solution exchange dynamics in flow reactors for liquid-phase transmission electron microscopy," *Nat. Commun.*, **2024**, 15, 2522

[2] D. L. Wise and G. Houghton, "The diffusion coefficients of ten slightly soluble gases in water at 10-60°C,", *Chem. Eng. Sci*., **1966**, 21, 999

[3] D. L. Wise and G. Houghton, "Diffusion coefficients of neon, krypton, xenon, carbon monoxide and nitric oxide in water at 10-60°C," *Chem. Eng. Sci*., **1968**, 23, 1211

[4] S. P. Cadogan, G. C. Maitland, and J. P. Martin Trusler, "Diffusion coefficients of CO_2 and N_2 in water at temperatures between 298.15 K and 423.15 K at pressures up to 45 MPa," *J. Chem. Eng. Data*, **2014**, 59, 519
